# Risk of Incident Diabetes in Relation to Long-term Exposure to Fine Particulate Matter in Ontario, Canada

**DOI:** 10.1289/ehp.1205958

**Published:** 2013-04-26

**Authors:** Hong Chen, Richard T. Burnett, Jeffrey C. Kwong, Paul J. Villeneuve, Mark S. Goldberg, Robert D. Brook, Aaron van Donkelaar, Michael Jerrett, Randall V. Martin, Jeffrey R. Brook, Ray Copes

**Affiliations:** 1 Public Health Ontario, Toronto, Ontario, Canada; 2 Dalla Lana School of Public Health, University of Toronto, Toronto, Ontario, Canada; 3 Population Studies Division, Health Canada, Ottawa, Ontario, Canada; 4 Institute for Clinical Evaluative Sciences, Toronto, Ontario, Canada; 5 Department of Family and Community Medicine, University of Toronto, Toronto, Ontario, Canada; 6 Department of Medicine, McGill University, Montreal, Quebec, Canada; 7 Division of Clinical Epidemiology, McGill University Health Centre, Montreal, Quebec, Canada; 8 Division of Cardiovascular Medicine, University of Michigan Medical School, Ann Arbor, Michigan, USA; 9 Department of Physics and Atmospheric Science, Dalhousie University, Halifax, Nova Scotia, Canada; 10 Division of Environmental Health Sciences, School of Public Health, University of California, Berkeley, Berkeley, California, USA; 11 Harvard–Smithsonian Centre for Astrophysics, Cambridge, Massachusetts, USA; 12 Air Quality Research Division, Environment Canada, Toronto, Ontario, Canada

**Keywords:** cohort study, diabetes, particulate air pollution

## Abstract

Background: Laboratory studies suggest that fine particulate matter (≤ 2.5 µm in diameter; PM_2.5_) can activate pathophysiological responses that may induce insulin resistance and type 2 diabetes. However, epidemiological evidence relating PM_2.5_ and diabetes is sparse, particularly for incident diabetes.

Objectives: We conducted a population-based cohort study to determine whether long-term exposure to ambient PM_2.5_ is associated with incident diabetes.

Methods: We assembled a cohort of 62,012 nondiabetic adults who lived in Ontario, Canada, and completed one of five population-based health surveys between 1996 and 2005. Follow-up extended until 31 December 2010. Incident diabetes diagnosed between 1996 and 2010 was ascertained using the Ontario Diabetes Database, a validated registry of persons diagnosed with diabetes (sensitivity = 86%, specificity = 97%). Six-year average concentrations of PM_2.5_ at the postal codes of baseline residences were derived from satellite observations. We used Cox proportional hazards models to estimate the associations, adjusting for various individual-level risk factors and contextual covariates such as smoking, body mass index, physical activity, and neighborhood-level household income. We also conducted multiple sensitivity analyses. In addition, we examined effect modification for selected comorbidities and sociodemographic characteristics.

Results: There were 6,310 incident cases of diabetes over 484,644 total person-years of follow-up. The adjusted hazard ratio for a 10-µg/m^3^ increase in PM_2.5_ was 1.11 (95% CI: 1.02, 1.21). Estimated associations were comparable among all sensitivity analyses. We did not find strong evidence of effect modification by comorbidities or sociodemographic covariates.

Conclusions: This study suggests that long-term exposure to PM_2.5_ may contribute to the development of diabetes.

Diabetes mellitus and its associated macrovascular (Kannel and McGee 1979; Manson et al. 1991a) and microvascular (Watkins and Edmonds 1983) complications constitute a serious threat to global human health and welfare (Wild et al. 2004). The burden of diabetes relates particularly to type 2 diabetes, which accounts for 90–95% of cases globally (American Diabetes Association 2012). Although obesity (Colditz et al. 1995), diet (Hu et al. 2001), and physical inactivity (Manson et al. 1991b) have been identified as important risk factors for type 2 diabetes, there is increasing evidence that insulin resistance, the underlying hallmark and pathophysiological mechanism leading to type 2 diabetes, can be aggravated by factors that promote inflammatory responses (Hotamisligil 2006).

It is only recently that ambient air pollution has been implicated in the etiology of type 2 diabetes (Brook et al. 2008). Exposure to air pollution has been associated with cardiovascular-related mortality and morbidity (Brook et al. 2010; Chen et al. 2008). There is also evidence that persons with diabetes are particularly susceptible to the acute effects of air pollution (Goldberg et al. 2006; O’Neill et al. 2005). In a mouse model, exposure to fine particulate matter (particles with an aerodynamic diameter ≤ 2.5 µm; PM_2.5_) increased blood glucose and induced adipose inflammation and insulin resistance (Sun et al. 2009). This animal study provides a potential biological basis for the link between air pollution and diabetes.

Because of the ubiquitous nature of exposure to air pollution, even a modest effect of air pollution on increasing the risk of diabetes may pose a large public health burden. To date, only four epidemiological studies have investigated the relationship between air pollution and incident diabetes (Andersen et al. 2012; Coogan et al. 2012; Krämer et al. 2010; Puett et al. 2011). Two reported positive associations between incident diabetes and PM_2.5_ and traffic-related pollutants such as nitrogen oxides (NO_x_) and nitrogen dioxide (NO_2_) (Coogan et al. 2012; Krämer et al. 2010). However, another study reported no association between incident diabetes and PM_2.5_ (Puett et al. 2011), and the fourth reported only a small increase in diabetes associated with NO_2_ (Andersen et al. 2012). Of the four cohort studies, one was based on a general population (Andersen et al. 2012), two included women only (Coogan et al. 2012; Krämer et al. 2010), and one comprised female nurses and male health professionals (Puett et al. 2011).

Given that few epidemiological studies have examined associations between air pollution and diabetes, and because little is known about the association in the general population, we conducted a population-based cohort study of long-term exposure to PM_2.5_ and incident diabetes in Ontario, Canada.

## Materials and Methods

*Study design and population*. The study was designed as a follow-up of respondents from Ontario, Canada, to the 1996/1997 cycle of National Population Health Survey of all Canadians (Statistics Canada 2010a) and the 2000/2001, 2002, 2003, and 2005 cycles of the Canadian Community Health Surveys among Canadians ≥ 12 years of age (Statistics Canada 2010b). These population-based surveys collected information related to health status, health care utilization, and determinants of health for the Canadian population in all provinces and territories, excluding full-time members of the Canadian military, institutional residents, and individuals living on Indian Reserves, Crown Lands, and certain remote regions. The same questions were used across surveys. The response rates of the surveys in Ontario varied from 73.4 to 92.8%, depending on the year (Statistics Canada 2010a, 2010b). The surveys have been used in health research, such as estimating the burden of cardiovascular diseases in Canada (Manuel et al. 2003).

The study population for the present analysis comprised all respondents who, at the time of survey, resided in Ontario, were ≥ 35 years of age, were registered with Ontario’s provincial health insurance plan, provided informed consent to share and link their responses to provincial health administrative data, and were free of diabetes. In addition, we restricted our analyses to Canadian-born individuals, because immigrants to Canada have unknown prior exposures and tend to have better health and health behaviors (McDonald and Kennedy 2004), but are more likely to live in areas with higher ground-level concentrations of PM_2.5_ than those born in Canada (Villeneuve et al. 2011). A total of 62,012 participants were included in the study and they were followed-up from the time of survey until 31 December 2010.

The study was approved by the Research Ethics Board of Sunnybrook Health Sciences Centre in Toronto, Ontario, Canada.

*Ascertainment of diabetes and other comorbidities*. We used the Ontario Diabetes Database (Toronto, ON, CA), a validated registry of diabetics in Ontario, to identify cohort members with and without diabetes (Hux et al. 2002; Lipscombe and Hux 2007). This database was developed using hospital discharge abstracts from the Canadian Institute for Health Information and physician service claims from the Ontario Health Insurance Plan database (Toronto, ON, CA). The hospital discharge database captures all hospital admissions among Ontario residents, whereas the claims database includes claims from approximately 98% of Ontario physicians because of the universal nature of the provincial medicare system (Chan 2000; Lipscombe and Hux 2007).

Individuals were entered in the diabetes database if they had at least one hospital admission with a diagnosis of diabetes [*International Classification of Diseases, 9th Revision, Clinical Modification* (ICD-9CM; Centers for Disease Control and Prevention 2012) diagnostic code 250 or *10th Revision* (ICD-10; World Health Organization 1993) code E10–E14 after 2002] or two or more physician claims for diabetes (code 250) within a 2-year period (Hux et al. 2002). Gestational diabetes was excluded. This algorithm has been validated using chart review and shown to have high sensitivity (86%) and specificity (97%) for identifying persons with diabetes (Hux et al. 2002). More recently, a study evaluating medical records of 22 family practitioners and their patients in southwestern Ontario confirmed the high sensitivity and specificity of this algorithm (Harris et al. 2010). Once included in the database, individuals remain in it until death or termination of Ontario health coverage. The eligibility of cohort members for health insurance and their vital status through the follow-up period were assessed using data from the Registered Persons Database (Toronto, ON, CA), a registry of all Ontario residents who have a health insurance number.

We linked the 62,012 cohort members to the diabetes database using encrypted health insurance numbers. We defined incident diabetes as cases diagnosed between the time of entry into the cohort and the end of follow-up (31 December 2010). Prevalent cases of diabetes diagnosed before the baseline survey were excluded from the analyses.

In addition, we determined whether participants had any of the following comorbidities at baseline: hypertension, congestive heart failure, acute myocardial infarction, asthma, and chronic obstructive pulmonary disease (COPD). These five comorbidities are often present in diabetic patients (Drury 1983; Fabbri and Rabe 2007; Mannino et al. 2008). We ascertained the five comorbidities using validated registries based on hospital discharge abstracts and physician service claims in Ontario [see Supplemental Material, Comorbidity Ascertainment, pp. 3–4 (http://dx.doi.org/10.1289/ehp.1205958)] (Gershon et al. 2009a, 2009b; Lee et al. 2003; Tu JV et al. 2001; Tu K et al. 2008).

*Assessment of long-term exposure to PM_2.5_*_._ To assess long-term exposure to ambient air pollution, we used satellite-based estimates of surface concentrations of PM_2.5_ (van Donkelaar et al. 2010). The satellite-based concentrations were derived from aerosol optical depth data from the Moderate Resolution Imaging Spectroradiometer (MODIS) and Multiangle Imaging Spectroradiometer (MISR) instruments onboard the National Aeronautics and Space Administration (NASA)’s Terra satellite (van Donkelaar et al. 2010). Using satellite data collected from 1 January 2001 through 31 December 2006, we derived long-term average concentrations of PM_2.5_ at a resolution of approximately 10 × 10 km and covered all North America below 70°N, which includes all of Ontario. These satellite-based long-term average concentrations of PM_2.5_ have been shown to correlate well with ground measurements at fixed-site stations across North America (Pearson correlation coefficient *r* = 0.77, *n* = 1057) (van Donkelaar et al. 2010). These satellite-based estimates have been applied previously to estimate associations between long-term exposure to air pollution and mortality (Crouse et al. 2012; Villeneuve et al. 2011) and to estimate the global burden of illnesses due to air pollution (Brauer et al. 2012). The remote sensing methodology used to estimate ground-level ambient concentrations of PM_2.5_ have been described in detail elsewhere (van Donkelaar et al. 2010). We assigned exposure by linking the exposure surface concentrations of PM_2.5_ to subjects’ residences at cohort entry using six-character postal codes. Six-character postal codes in urban areas represent the centroid of the blocks in which the cohort members lived.

*Potential confounding variables*. From the self-reported health surveys, we extracted information on marital status, race/ethnicity (white, black, Asian, Arab, Latin American, other), education attainment (less than high school, high school, some postsecondary, and university), and household income adequacy (lowest income, lower middle income, middle income, upper middle income, and upper income). Household income adequacy is an index used by Statistics Canada that accounts for total household income and household size (Statistics Canada 2010a, 2010b). Because 98% of the cohort was classified as white, we dichotomized race/ethnicity as white or nonwhite.

In addition, we extracted baseline survey data for height and weight to calculate body mass index (BMI; kilograms per meter squared). We also obtained information on smoking status (never smoker, current smoker, former smoker), alcohol consumption (more than once a month, less than once a month, former drinker, never drank), daily consumption of fruits and vegetables (< 5 servings/day, ≥ 5 servings/day), physical activity (≥ 3.0, 1.5–2.9, < 1.5 kcal/kg/day of energy expenditure for leisure activities), and urban/rural residence. Urban areas are those continuously built-up areas having a population ≥ 1,000 and a population density ≥ 400/km^2^ based on current census population counts (Statistics Canada 2010a, 2010b).

Using the Canadian Census data for 1996, 2001, and 2006 (Statistics Canada 2012a, 2012b, 2012c), we created contextual variables at the census tract level for the proportion of population ≥ 15 years of age with less than high school education, unemployment rate, and mean household income. Because census tracts are not defined for rural areas, we derived these three variables for rural residents according to their census subdivision, which is the next higher geographic unit, usually representing a municipality or equivalent. We assigned the values of contextual variables derived from the 1996/1997 census for individuals who entered the cohort in 1996; from the 2001 census for individuals who entered in 2000/2001, 2002, or 2003; and from the 2006 census for individuals who entered in 2005. Census tracts are small and relatively homogeneous geographic units that usually comprise a population of 2,500–8,000.

To control for regional-scale spatial patterns in the incidence of diabetes that could be explained by factors other than pollution, we created a dichotomous indicator variable classifying Ontario into southern and northern regions based on the 14 Ontario Local Health Integrated Networks. The Local Health Integrated Networks are responsible for planning, integrating, and funding various local health care services in Ontario.

*Statistical analysis*. We used a stratified Cox proportional hazards model with strata defined as single-year age groups, cycle of survey, and region (south/north). We excluded 1,936 individuals with missing data for BMI, and we created for all other covariates a separate category for missing values, leaving 60,076 cohort members in all analyses.

The outcome was the incident diagnosis date of diabetes as indicated in the diabetes database. Follow-up time was measured in days, calculated from the date of interview until the date of incident diabetes, or death, or were no longer eligible for provincial health insurance, or until the end of follow-up (31 December 2010).

We modeled associations between PM_2.5_ and incident diabetes, adjusting for sex, marital status, education, household income adequacy, race/ethnicity, BMI (a linear term and a quadratic term), physical activity, smoking, drinking, diet, urban residency, hypertension at baseline, area-level unemployment, education, and mean household income at baseline. In separate analyses we also controlled for other comorbidities including congestive heart failure, acute myocardial infarction, COPD, and asthma.

We routinely tested for deviations from the proportional hazards assumption by assessing whether the cross-product of each variable with the natural logarithm of the time variable was statistically significant (α = 0.05). We also verified the assumption of linearity for PM_2.5_ and all other continuous variables by using natural cubic spline functions with two and three degrees of freedom. We examined plots of the concentration–response curves and used the Akaike Information Criterion (AIC) to assess the relative goodness of fit for these models. A difference of > 4 AIC points is considered to prefer one model over another (Leffondre et al. 2002). Because there was no evidence of departure from linearity for the relation of PM_2.5_ and diabetes, we report adjusted hazard ratios (HR) and 95% CIs for every 10-µg/m^3^ increase of PM_2.5_ (referred to as HR_10_). We chose an increment of 10 µg/m^3^ to facilitate comparisons of our findings with other studies (Coogan et al. 2012; Puett et al. 2011).

In addition, we investigated potential effect modification by age, sex, BMI, education, race/ethnicity, household income adequacy, physical activity, smoking, and comorbidities by assessing whether the interaction term that was the cross-product of each variable with PM_2.5_ value was statistically significant.

*Sensitivity analyses*. Our main analysis focused on exposure assigned according to residential postal codes at cohort entry. To assess the impact of residential mobility on the effect estimates, we performed three sensitivity analyses by restricting the follow-up period to first 2 or 5 years, respectively; restricting to 55,708 participants who had lived at their baseline address for at least 5 years before baseline; and modeling time-weighted exposure since cohort entry until the event, with weights for each participant defined by the time spent at each address. For the latter analysis, we obtained residence for each subject and each year from the Registered Persons Database (Toronto, ON, CA) for the period 1996–2010, and derived annual estimates of PM_2.5_ exposure by assigning the 6-year mean concentration of PM_2.5_ at each annual postal code.

We also performed additional sensitivity analyses by restricting to participants who had used health services 1–2 years before the baseline because of a concern that the frequency of health care utilization might influence the likelihood of detecting diabetes; excluding participants with missing information on diet because two of the five surveys (1996 and 2002; 27% of the study population) did not include diet-related questions; excluding participants from the 2002 cycle of health survey because this survey had the lowest response rate (73.4%); and restricting the analysis to southern Ontario, where 83% of the cohort lived.

We next investigated whether the association might be influenced by spatial dependence among study subjects. We fitted the Cox model with a frailty (random effect) term for Ontario Local Health Integration Networks to allow for the possibility that the effect estimate for diabetes may vary from network to network in the estimation of the main effect and its variance. A gamma distribution for the frailties was assumed, with an exchangeable correlation structure within network. We compared models with and without a frailty term using the AIC. We repeated this analysis by using a frailty term for grids from PM_2.5_ exposure surface (10 × 10 km) as a random effect.

Furthermore, we examined whether associations changed over time by additionally adjusting the Cox model for annual mean concentration of PM_2.5_ in Ontario (as a linear term) for the period 1996–2010. We derived the long-term trend of PM_2.5_ using data from all air quality monitoring stations across Ontario that operated for at least half of the study period [see Supplemental Material, Figure S1 (http://dx.doi.org/10.1289/ehp.1205958)]. We also tested for interactions between time periods (1996–2000 and 2007–2010) and PM_2.5_.

## Results

The cohort comprised 484,644 person-years of observations. Mean (± SD) follow-up was 8 ± 3.2 years. The mean age of the cohort at time of entry was 54.9 years (Table 1). Forty–five percent of the cohort members were men, 63% were married, 24% were current smokers, and 54% were either overweight or obese (BMI ≥ 25 kg/m^2^). In addition, 29% of the cohort had hypertension, 10% had COPD, and 3% had congestive heart failure at baseline. Average unemployment among the census tracts was 7%, and mean household income was approximately Can$62,300.

**Table 1:** Baseline characteristics of study population (*n* = 62,012).

Baseline characteristics	Mean ± SD or percent
Individual risk factors	
Age (years)	54.9±14.2
Men	45
Marital status	
Married	63
Single	11
Separated, widowed, or divorced	26
Race	
White	98
Nonwhite	1
Missing	1
BMI (kg/m^2^)	26.2±4.7
<18.5	2
18.5–24.9	41
25.0–29.9	37
≥30	17
Missing	3
Education	
< High school	22
High school	18
Beyond high school	58
Missing	2
Annual household income adequacy^*a*^	
Lowest income quintile	3
Lower-middle income quintile	7
Middle income quintile	18
Upper-middle income quintile	32
Upper income quintile	31
Missing	9
Smoking status	
Never smoker	27
Current smoker	24
Former smoker	41
Missing	8
Alcohol consumption^*b*^	
Regular drinker	62
Occasional or former drinker	34
Never drinker	4
Total daily consumption of fruits and vegetables	
<5 times/servings/day	44
≥5 times/servings/day	29
Missing	27
Energy expenditure (kcal/kg/day)^*c*^	
≥3.0 (active)	22
1.5–2.9 (moderate)	25
<1.5 (inactive)	51
Missing	2
Preexisting comorbidity	
Hypertension	29
Acute myocardial infarction	2
Congestive heart failure	3
COPD	10
Asthma	9
Proportion of cohort lived in an urban area^*d*^	66
Proportion of cohort lived in southern region	83
Area-level risk factors^*e*^**	
Percentage ≥15 years of age with less than high school education	28
Percentage ≥15 years of age without employment	7
Average household income (Can$1,000)	62.3±17.5
^***a***^Index used by Statistics Canada (2010a, 2010b) that accounts for total household income and household size. ^***b***^Regular drinker: ≥once each month; occasional drinker: <once each month; former drinker: ever had a drink. ^***c***^Average daily energy expenditure during leisure activities based on the frequency and duration of each activity and the estimated metabolic energy cost expressed as a multiple of the resting metabolic rate. ^***d***^Urban areas are defined by Statistics Canada (2010a, 2010b) as continuously built-up areas with a population of ≥1,000 and a population density of ≥400/km^2^. To be considered as continuous, the built-up area must not have a discontinuity >2km. All other areas were considered rural. ^***e***^From Canadian Census, at the census tract level (Statistics Canada 2012b).	

**Figure 1:**
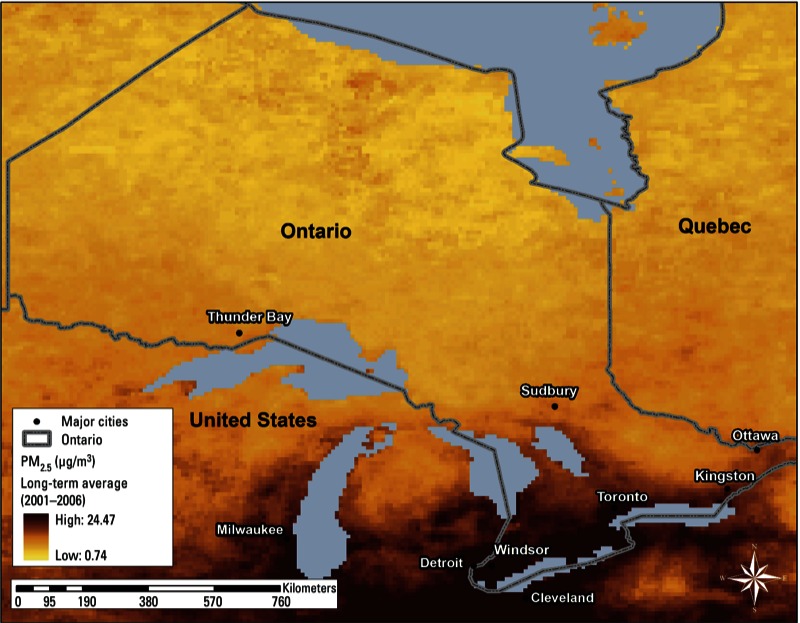
Mean satellite-derived estimates of PM_2.5_ across Ontario, Canada, 2001–2006.

Of the cohort, 17%, 26%, 8%, 25%, and 24% were enrolled from the surveys of 1996/1997, 2000/2001, 2002, 2003, and 2005, respectively. Among cohort members of the five surveys, we identified 1,503, 1,697, 495, 1,474, and 1,141 incident cases of diabetes during the follow-up, with a total of 6,310 cases. Average estimated exposure to PM_2.5_ during 2001–2006 was 10.6 µg/m^3^ (range, 2.6–19.1 µg/m^3^), with the highest average concentrations in southern Ontario (Figure 1).

*Associations between diabetes and PM_2.5_*_._ For every 10-µg/m^3^ increase of PM_2.5_, the hazard ratio for incident diabetes (HR_10_) was 1.08 (95% CI: 0.99, 1.17) adjusting for sex and stratifying on age, survey year, and region (Table 2). Adjustment for education, household income, and BMI strengthened the association between PM_2.5_ and diabetes (HR_10_ -~⊇1.12). Additionally controlling for physical activity, smoking, and other individual-level covariates did not appreciably change the HR. In the fully adjusted model that included all individual-level covariates, contextual covariates, and comorbid conditions, the HR_10_ was 1.11 (95% CI: 1.02, 1.21). Modeling PM_2.5_ using natural splines did not improve model fit according to AIC relative to the model that assumed linearity for PM_2.5_ (AIC = 57441 for the model with a linear term of PM_2.5_; AIC = 57442 for the model with natural spline), suggesting a potential log-linear relationship between PM_2.5_ and incident diabetes [see Supplemental Material, Figure S2 (http://dx.doi.org/10.1289/ehp.1205958)].

**Table 2:** HRs (95% CIs) for the association between incident diabetes and a 10‑µg/m^3^ increase in PM_2.5_.

	HR (95% CI)
Adjusting for sex and stratified by age, survey year, and region	1.08 (0.99, 1.17)
+ All individual-level covariates^*a*^	1.11 (1.02, 1.21)
+ All neighborhood-level covariates^*b*^	1.11 (1.02, 1.21)
+ All other comorbidities^*c*^	1.11 (1.02, 1.21)
^***a***^Adjusted for sex, marital status, education, household income adequacy, BMI, physical activity, smoking, alcohol consumption, diet, race, hypertension, and urban residency. ^***b***^Also adjusted for neighborhood-level ­unemployment rate, education, and household income. ^***c***^Also adjusted for COPD, asthma, congestive heart ­failure, and acute myocardial infarction.	

We observed larger HRs between diabetes and PM_2.5_ among 819 participants with preexisting COPD (HR_10_ = 1.33; 95% CI: 1.03, 1.71 compared with HR_10_ = 1.08; 95% CI: 0.98, 1,18 for those without COPD; *p*-interaction = 0.13), and among women (HR_10_ = 1.17; 95% CI: 1.03, 1.32 compared with HR_10_ = 1.03; 95% CI: 0.91, 1.16 for men; *p*-interaction = 0.15), and participants < 50 or > 65 years of age compared with 50–65 years (*p*-interaction = 0.19) (Table 3).

**Table 3:** HRs^*a*^ (95% CIs) for the associations of incident diabetes with a 10‑µg/m^3^ increase in PM_2.5_, by selected characteristics.

Characteristic	No. of cases	HR (95% CI)	*p*-Value for interaction with PM_2.5_^*b*^
Age (years)			0.19
<50	1,690	1.19 (1.00, 1.40)	
50–65	2,649	1.00 (0.88, 1.15)	
>65	1,971	1.18 (1.01, 1.38)	
Sex			0.15
Men	3,239	1.03 (0.91, 1.16)	
Women	3,071	1.17 (1.03, 1.32)	
BMI (kg/m^2^)			0.63
<25.0	1,365	1.20 (1.00, 1.45)	
25.0–29.9	2,501	1.08 (0.94, 1.25)	
≥30.0	2,415	1.08 (0.94, 1.25)	
Education			0.68
≤ High school	3,085	1.13 (1.00, 1.28)	
Beyond high school	3,137	1.09 (0.96, 1.23)	
Race			0.49
White	6,145	1.11 (1.01, 1.20)	
Nonwhite	127	0.79 (0.31, 2.03)	
COPD			0.13
Yes	819	1.33 (1.03, 1.71)	
No	5,491	1.08 (0.98, 1.18)	
Congestive heart failure			0.84
Yes	231	1.16 (0.66, 2.04)	
No	6,097	1.09 (1.00, 1.19)	
Hypertension			0.51
Yes	2,882	1.08 (0.95, 1.23)	
No	3,428	1.14 (1.02, 1.29)	
Acute myocardial infarction			0.92
Yes	209	1.05 (0.52, 2.16)	
No	6,101	1.11 (1.00, 1.20)	
Asthma			0.66
Yes	666	1.04 (0.79, 1.37)	
No	5,644	1.11 (1.01, 1.22)	
^***a***^Separate models stratified by age, survey year, and region, and adjusted for sex, marital status, education, household income, BMI, physical activity, smoking, alcohol consumption, diet, hypertension, race, urban residency, neighborhood-level unemployment rate, neighborhood-level education, and neighborhood-level household income. ^***b***^Likelihood ratio test.			

*Sensitivity analyses*. Restricting the analysis to the first 2 and 5 years of follow-up slightly increased the effect estimates for diabetes (Table 4). Using time-weighted exposure as an alterative exposure metric did not result in appreciable difference in the estimate of association. In addition, the risk estimate was insensitive to exclusion of participants who had recently moved to their baseline addresses before the study, those who had not used health services within the previous 2 years, those who enrolled in the 2002 survey, or those who lived in northern Ontario. Additionally, we excluded participants with missing information on diet, yielding an HR_10_ of 1.10 (95% CI: 0.96, 1.23). Furthermore, adding a frailty term to allow for random effects according to Ontario Local Health Integration Networks or grids from PM_2.5_ exposure surface had little impact on the HR or its variance. Last, there was no strong evidence of variation in the estimated effects of PM_2.5_ over time, and the test for interactions between periods and PM_2.5_ was not statistically significant (*p*-interactions = 0.95 and 0.35).

**Table 4:** Sensitivity analyses^*a*^ for the associations of incident diabetes with every 10‑µg/m^3^ increase of PM_2.5_.

Sensitivity analysis	No. of cases	HR (95% CI)
Restricted to follow-up period within		
First 2 years since the time of entry	2,087	1.15 (0.98, 1.33)
First 5 years since the time of entry	4,291	1.13 (1.01, 1.26)
Modeled time-weighted exposure	6,300	1.10 (1.01, 1.20)
Restricted to participants who lived in their baseline addresses for at least 5 years before cohort entry	5,198	1.12 (1.01, 1.23)
Restricted to participants who had ≥1 health care contact^*b*^ within		
Previous year	5,905	1.11 (1.01, 1.21)
Previous 2 years	6,134	1.10 (1.01, 1.21)
Restricted to participants who lived in southern Ontario	5,108	1.08 (1.00, 1.19)
Excluded participants from the 2002 survey	5,805	1.12 (1.02, 1.23)
Adjusted for annual mean concentration of PM_2.5_ across Ontario, 1996–2010	6,300	1.11 (1.02, 1.21)
Added a frailty term (random effect) to investigate spatial dependence as a source of bias		
+ frailty term for Ontario Local Health Integration Networks^*c*^	6,300	1.12 (1.02, 1.22)
+ frailty term for grids from the exposure surface of PM_2.5_ (10×10 km)	6,300	1.11 (1.01, 1.21)
^***a***^Model stratified by age, survey year and region, and adjusted for sex, marital status, education, household income, BMI, physical activity, smoking, alcohol consumption, diet, race, hypertension, urban residency, neighborhood-level unemployment rate, education, household income, and COPD, asthma, congestive heart failure, and acute myocardial infarction. ^***b***^Health care contact is defined as having an Ontario Health Insurance Plan claim, Ontario Drug Benefit claim, hospitalization record, same-day surgery record, ambulatory care, chronic care service, home care service, inpatient rehabilitation, or inpatient mental health care. ^***c***^There are a total of 14 Local Health Integration Networks in Ontario.		

## Discussion

In this population-based cohort study of 62,012 adults in Ontario, we found that long-term exposure to PM_2.5_ was associated with an increased risk of incident diabetes after controlling for various individual and neighborhood covariates. The estimate of association was insensitive to various sensitivity analyses. Additionally, we did not find strong evidence for effect modification by selected comorbidities and sociodemographic covariates.

This study provides evidence for the association between incident diabetes and PM_2.5_. Few studies have investigated the relationship between incident diabetes and air pollution. In a study of 1,775 women in the Ruhr district of Germany, Krämer et al. (2010) reported an adjusted HR of 1.27 (95% CI: 1.09, 1.48) for incident diabetes for every interquartile-range (IQR) increase of PM_2.5_ (IQR = 0.4/10^5^ m using absorbance-based measurement) and 1.42 (95% CI: 1.16, 1.73) per IQR of NO_2_ (IQR = 8 ppb). A second study of 4,204 African-American women in Los Angeles, California, reported an HR of 1.25 (95% CI: 1.07, 1.46) per IQR of NO_x_ (12.4 ppb) and 1.63 (95% CI: 0.78, 3.44) for a 10-µg/m^3^ increase in PM_2.5_ (Coogan et al. 2012).

In contrast, there was little evidence of an association between incident diabetes and particulate pollutants [PM_2.5_, PM_2.5–10_, PM_10_ (PM with diameter 2.5–10 µm and ≤ 10 µm)] in a cohort of female nurses (*n* = 74,412) or a cohort of male health professionals (*n* = 15,048) living in metropolitan areas of the northeastern and midwestern United States, though residential proximity to roadways was associated with diabetes among the nurses (0–49 m vs. ≥ 200 m: HR = 1.14; 95% CI: 1.03, 1.27) (Puett et al. 2011). In a study of 51,818 participants in Denmark (Andersen et al. 2012), an IQR increase in NO_2_ (2.6 ppb) was not associated with all cases of diabetes (HR = 1.00; 95% CI: 0.97, 1.04), but was associated with cases who were identified using a more strict case definition (HR = 1.04; 95% CI: 1.00, 1.08). Finally, there was a 4% increase in the adjusted odds (95% CI: 0%, 8%) of diabetes prevalence with each parts per billion increase of NO_2_ among women in a cross-sectional study of respiratory clinic patients in Hamilton and Toronto, Ontario, but there was no association among men (Brook et al. 2008).

Although associations with diabetes in previous studies were relatively consistent for NO_2_, associations with PM_2.5_ were not. The inconsistency may be attributable to chance, differences in population characteristics, misclassification of exposure (resulting from different exposure methods across studies), or inherent differences in the toxicological properties of the pollutants. In these prior studies, positive associations were reported mostly among women (Coogan et al. 2012; Krämer et al. 2010; Puett et al. 2011), and we also found a stronger association among women than men. This stronger association could be attributable to chance, but it may also reflect smaller errors in exposure assessment for women, because women tend to spend more time in and around home than men (Brook et al. 2008). Physiological and lifestyle differences may also contribute to the difference in effect estimates between men and women (Brook et al. 2008). Associations also varied by age, with stronger effects of PM_2.5_ among those above and below 50–65 years of age at baseline. However, our data are inadequate to investigate this pattern further.

Although increasing BMI is a strong risk factor for diabetes (Colditz et al. 1995), our results do not support the idea that being overweight or obese would enhance susceptibility to the effects of air pollution on diabetes. We did not find strong evidence suggesting that other comorbidities materially altered the association between PM_2.5_ and diabetes, though power to detect differences was limited. Because inflammation is a key feature of common chronic diseases such as COPD (Rana et al. 2004) and an important pathophysiological response to PM_2.5_ exposure, further investigation is needed on whether the chronic state of inflammation in patients with these conditions may heighten their susceptibility to diabetes as a consequence of PM_2.5_ exposure.

To our knowledge, this is the largest study of incident diabetes in a population-based cohort to date. According to the Canadian Census in 2001 (Statistics Canada 2012b), this cohort is representative of the Canadian-born population ≥ 35 years of age in Ontario (mean age = 53.3, men = 48%, married = 65%, and white = 99%). We obtained extensive individual information on known risk factors, and the diagnosis of diabetes was based on a validated registry with very high sensitivity and specificity (Hux et al. 2002). Finally, the use of satellite-based long-term average estimates of PM_2.5_ ensures virtually complete spatial coverage of PM_2.5_ among all cohort members. The satellite-based estimates have been shown to correlate well with ground-based measurements (van Donkelaar et al. 2010). The ambient level of PM_2.5_ in Ontario (annual mean in 2000, 11.2 µg/m^3^) was much lower than average exposures in previous studies conducted in Los Angeles, California (annual mean PM_2.5_ in 2000, 20.7 µg/m^3^) (Coogan et al. 2012) and in the Ruhr district of Germany (annual mean PM_2.5_ in 2002, 22.4 µg/m^3^) (Krämer et al. 2010).

This study has several limitations that should be considered. First, we could not differentiate between type 1 and type 2 diabetes. However, given that type 2 diabetes accounts for > 90% of all diabetes cases globally and that all cohort members were ≥ 35 years of age at entry (mean, ~ 55 years), the vast majority of incident diabetes in this cohort is expected to be type 2 diabetes (American Diabetes Association 2012).

Second, we could not identify undiagnosed cases of diabetes in the cohort. Although incomplete diagnosis of cohort members is a potential limitation of this study, effect estimates were virtually unchanged when we restricted the analysis to subjects who had used health care services during the 1–2 years before baseline as a proxy indicator of health care utilization, which may be related to the diagnosis of diabetes. Because of universal health care in Ontario, it is expected that we may have underestimated the true effects because this measurement error was likely independent of the exposure.

Third, the spatial pattern in exposure used was derived for the period 2001 to 2006 only. However, the spatial gradients of ambient PM_2.5_ in Ontario remained stable during the follow-up period (1996–2010) and that variability in the concentrations of PM_2.5_ is primarily spatial in nature and not temporal [see Supplemental Material, p. 4 (http://dx.doi.org/10.1289/ehp.1205958)]. Studies conducted in diverse locations across the United States have also demonstrated long-term stability in the spatial patterns of PM_2.5_ (Jerrett et al. 2005; Miller et al. 2007; Pope et al. 2002). We therefore expect that the spatial contrasts in PM_2.5_ during 2001–2006 is a reasonable representation of longer-term spatial exposure to PM_2.5_ in Ontario. The spatial resolution of PM_2.5_ exposure surface (i.e., 10 × 10 km), however, meant that we were unable to estimate associations at finer spatial scale. We also did not have information on daily activity. To assess the impact of residential mobility, we performed various sensitivity analyses, which did not result in appreciable differences in risk estimates. Also, our analyses did not consider the mixture of air pollutants to which subjects may have been exposed.

Fourth, we did not have family history of diabetes or occupational exposure to dust/fumes in the surveys, and information on potential confounding variables was obtained at baseline only. Twenty-seven percent of the cohort had missing information on diet which was modeled as a separate category. However, there was little change in the association between PM_2.5_ and diabetes when we excluded members with missing information on diet.

This study suggests that long-term exposure to PM_2.5_ may contribute to the development of diabetes. A plausible biological mechanism linking exposure to PM_2.5_ with diabetes may be indirect effects mediated through systemic proinflammatory and oxidative responses (Brook et al. 2008). Sun et al. (2009) found that exposing mice to ambient PM_2.5_ at the concentration of 72 µg/m^3^ for 6 hr each day over 10 weeks exacerbated insulin resistance by enhancing systemic inﬂammatory response and inﬂammation in adipose tissue. Other plausible mechanisms include autonomic nervous system imbalance and endothelial dysfunction that may be triggered by PM_2.5_, which would in turn induce vasoconstriction and result in reduced insulin sensitivity (Brook et al. 2008; Coogan et al. 2012).

## Conclusions

In summary, we investigated the association between long-term exposure to PM_2.5_ and the risk of incident diabetes in a large cohort in Ontario, Canada. Results from this study support a possible relationship between PM_2.5_ and diabetes.

## Supplemental Material

(696 KB) PDF
