# Supplementary material for: Risk of Incident Diabetes in Relation to Long-term Exposure to Fine Particulate Matter in Ontario, Canada
Source: Environ Health Perspect. 2013 Apr 26;121(7):804–10. doi: 10.1289/ehp.1205958 (PMC3701997; doi:10.1289/ehp.1205958)
Supplement: (696 KB) PDF [file ehp.1205958.s001.pdf]

Supplemental Material

Risk of Incident Diabetes in Relation to Long-term Exposure to Fine Particulate Matter in Ontario, Canada

Hong Chen,[1,2] Richard T. Burnett,[3] Jeffrey C. Kwong,[1,4,5] Paul J. Villeneuve,[2,3] Mark S. Goldberg,[6,7] Robert D. Brook,[8] Aaron van Donkelaar,[9] Michael Jerrett,[10] Randall V. Martin,[9,11] Jeffrey R. Brook,[12] and Ray Copes[1,2]

[1] Public Health Ontario, Toronto, Ontario, Canada

[2]Dalla Lana School of Public Health, University of Toronto, Toronto, Ontario, Canada

[3]Population Studies Division, Health Canada, Ottawa, Ontario, Canada

[4]Institute for Clinical Evaluative Sciences, Toronto, Ontario, Canada

[5]Department of Family and Community Medicine, University of Toronto, Toronto, Ontario, Canada

[6]Department of Medicine, McGill University, Montreal, Quebec, Canada

[7]Division of Clinical Epidemiology, McGill University Health Centre, Montreal, Quebec, Canada

[8]Division of Cardiovascular Medicine, University of Michigan Medical School, Ann Arbor, Michigan, USA

[9]Department of Physics and Atmospheric Science, Dalhousie University, Halifax, Nova Scotia, Canada

[10]Division of Environmental Health Sciences, School of Public Health, University of California, Berkeley, California, USA

[11]Harvard-Smithsonian Centre for Astrophysics, Cambridge, Massachusetts, USA

[12]Air Quality Research Division, Environment Canada, Toronto, Ontario, Canada

Correspondence:

Hong Chen, PhD

Public Health Ontario

480 University Avenue, Suite 300

Toronto, Ontario M5G 1V2

Tel: 647-260-7109

Email: hong.chen@oahpp.ca

Table of Contents

Comorbidity Ascertainment ……………………………..…………………………………… 3

Long-term Stability of Satellite-based Six-year Mean Concentrations of $PM_{2.5}$ ….…………... 4

Comparison of Spatial Resolution for Different Datasets in the Study……………………….... 6

Figure S1. Trends in annual average concentrations of $PM_{2.5}$ (in μg/m^3) in six cities in Ontario, Canada between 1996 and 2010………………………………………………………….. 7

Figure S2. Concentration-response relationship between the concentration of $PM_{2.5}$ and incident diabetes among the cohort………………………………………………………………... 8

References……………………………………………………………………………….....

9

Comorbidity Ascertainment

- *Ontario Hypertension Database*
  - Data source: hospital discharge abstracts from Canadian Institute of Health Information (CIHI) (including same day surgery) database, physician service claims from Ontario Health Insurance Plan (OHIP) database
  - Time period covered: 1988 onward
  - Case definition: one hospital admission with a hypertension diagnosis, or an OHIP claim with a hypertension diagnosis followed within two years by either an OHIP claim or a hospital admission with a hypertension diagnosis (ICD-9: 401-405; ICD-10: I10-I13, I15).
  - Sensitivity=72%, specificity=95%, positive predictive value=87%, and negative predictive value=88% (Tu et al. 2008).
- *Ontario Asthma Database*
  - Data source: hospital discharge abstracts from CIHI database, physician service claims from OHIP database
  - Time period covered: 1991 onward
  - Case definition: one hospital admission with an asthma diagnosis or two OHIP claims with asthma diagnosis within a two-year period (ICD-9: 493; ICD-10: J45, J46).
  - Sensitivity=84% and specificity=76% in adults who are 18 years of age and older (Gershon et al. 2009a).
- *Ontario Congestive Heart Failure (CHF) Database*
  - Data source: CIHI discharge abstract database, physician service claims from the OHIP database, emergency department records from National Ambulatory Care Reporting System (NACRS)
  - Time period covered: 1991 onward
  - Case definition: one hospital admission with a CHF diagnosis or an OHIP claim/ emergency department record with a CHF diagnosis followed within two years by either

    a second OHIP claim/NACRS record or a hospital admission with a CHF diagnosis (ICD-9: 428; ICD-10: I500, I501, I509).
  - Sensitivity=85% and specificity=97% (Yeung et al. 2012).
- *Ontario Chronic Obstructive Pulmonary Disease (COPD) Database*
  - Data source: CIHI discharge abstract database, emergency department records from NACRS
  - Time period covered: 1991 onward
  - Case definition: one or more ambulatory claims and/or one or more hospitalizations for COPD (ICD-9: 491, 492, 496; ICD-10: J41, J42, J43, J44).
  - Sensitivity=85% and specificity=79% (Gershon et al. 2009b).
- *Ontario Myocardial Infarction Database (OMID)*
  - Data source: CIHI discharge abstract database
  - Time period covered: 1988 onward
  - Case definition: all patients with a most responsible diagnosis with ICD-9 410 or ICD-10 I21. Exclusion criteria are those who were not Ontario residents, who had a MI as a complication after admission to hospital, who were discharged with a total length of stay < 3 days, who were readmitted to hospital with a MI in the past year and those transferred from another acute care institution, or who were initially admitted to a noncardiac surgical service (Tu et al. 1999).
  - Sensitivity=89% and specificity=93% (Austin et al. 2002).

Assess Long-term Stability of the Satellite-based six-year Mean Concentrations of $PM_{2.5}$

We verified long-term stability in the spatial patterns of six-year average concentrations of $PM_{2.5}$ over study period. In doing this, we compiled historical data on the monitoring of $PM_{2.5}$ from Environment Canada's National Air Pollution Surveillance (NAPS) network (Environment Canada 2010). We excluded fixed-site monitors that were located outside Ontario and that were operated for less than half of the study period, leaving sufficient data to derive annual mean

concentrations for six cities in Ontario. These six cities are Toronto, Hamilton, Windsor, Ottawa, Simcoe, and St. Petre.

Using the monitoring data, we estimated for each city long-term average concentrations of $PM_{2.5}$ over the entire study period. We compared the long-term averages of $PM_{2.5}$ with the satellite-based six-year mean concentrations averaged among all study subjects in each city. Mean concentrations of $PM_{2.5}$ between the two periods were reasonably well correlated (Pearson's correlation coefficient $r = 0.77$).

Using the annual mean concentrations of $PM_{2.5}$ from the six cities, we further estimated the total variance of $PM_{2.5}$ across the six cities and throughout the study period between 1996 and 2010. In addition, we estimated the variance of $PM_{2.5}$ that was due to temporal variability from 1996 to 2010. This was done by calculating mean exposure averaged across the six cities for each year and then estimating the variance of the annual averages over time. The total variance was 6.70 $(\mu g/m^3)^2$ while the temporal variance was 2.25 $(\mu g/m^3)^2$. Thus, 67% of the total variation in the concentrations of $PM_{2.5}$ among the six cities between 1996 and 2010 is associated with spatial variability and only 33% with variation over time. This result suggests that variability in the concentrations of $PM_{2.5}$ in Ontario is primarily spatial in nature and not temporal. This finding is reinforced by the fact that the rank ordering of the six Ontario cities by relative levels of $PM_{2.5}$ during the study period remained nearly constant (Figure S1).

The representativeness of shorter-term $PM_{2.5}$ measurements for longer-term exposure has been reported in several previous studies (Jerrett et al. 2005; Miller et al. 2007; Pope et al. 2002). For example, in the American Cancer Society Cancer Prevention Study II (ACS study) that was conducted in Los Angels, California, Jerrett *et al.* (2005) assessed the relationship between $PM_{2.5}$

measured in 1980 and those in 1999-2000 at 51 fixed-site monitors. They found strong correlation in $PM_{2.5}$ measurements between the two periods (the coefficient of determination, R^2=61% or r=0.78), indicating that areas with higher particle concentrations in earlier periods were likely to retain their spatial ranking. Long-term stability in the spatial patterns of $PM_{2.5}$ has also been demonstrated in another ACS study that included entire ACS cohort from 116 metropolitan areas in the U.S.A (Pope et al. 2002) and in the Women's Health Initiative Study that comprised subjects from 36 metropolitan areas in the U.S.A (Miller et al. 2007). We therefore expect that the spatial contrast in $PM_{2.5}$ over 2001-2006 provided reasonable estimates of longer-term spatial exposure to $PM_{2.5}$ in Ontario.

Comparison of Spatial Resolution for Different Datasets in the Study

Spatial resolution for different datasets used in our study is described as follows: Postal codes (a total of 269,676 in Ontario) > Census tract (2,136 in Ontario) > 10km by 10km grids in the $PM_{2.5}$ exposure surface (1,198 grids in Ontario) > Census division (50 in Ontario) > Ontario local health integration networks or LIHN (a total of 14 in Ontario).

These datasets were created by different organizations for different purposes; as a result, their areas may overlap. For example, LIHNs may overlap with census divisions.

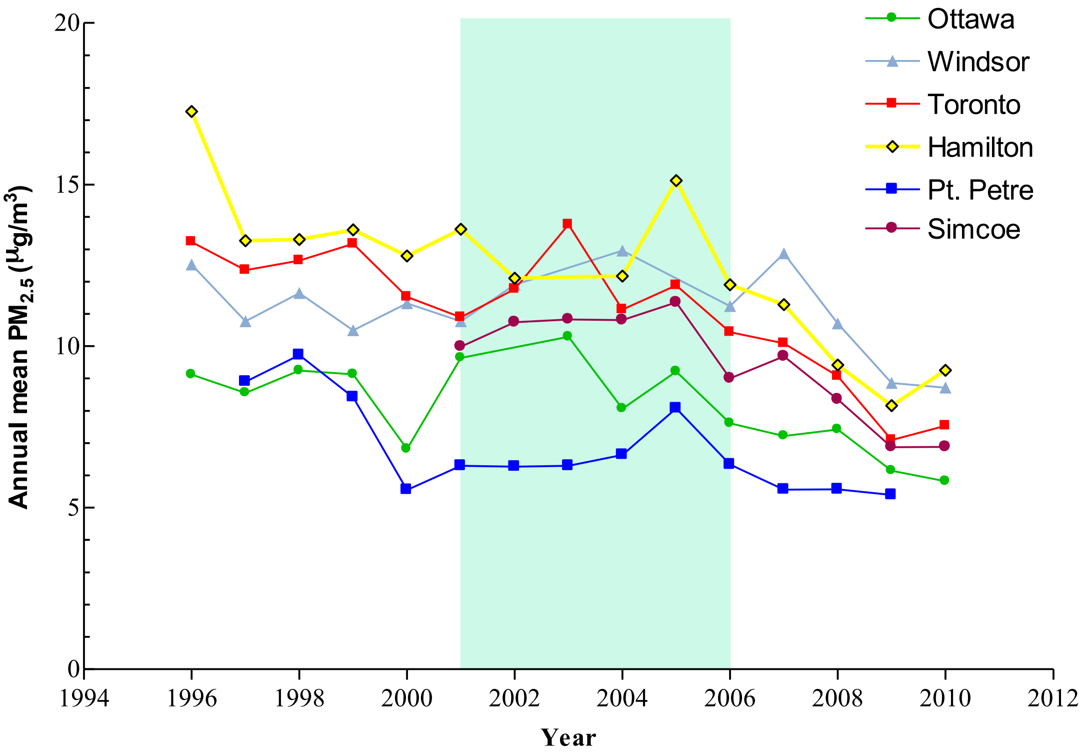

Supplemental Material, Figure S1. Trends in annual average concentrations of $PM_{2.5}$ (in μg/m^3) in six cities in Ontario, Canada between 1996 and 2010. Data were obtained from Environment Canada's National Air Pollution Surveillance (NAPS) network. Fixed-site monitors operated for less than half of the entire study period (<8 years) were excluded. The shaded area denotes the time period during which satellite-based surface measurements of $PM_{2.5}$ were available.

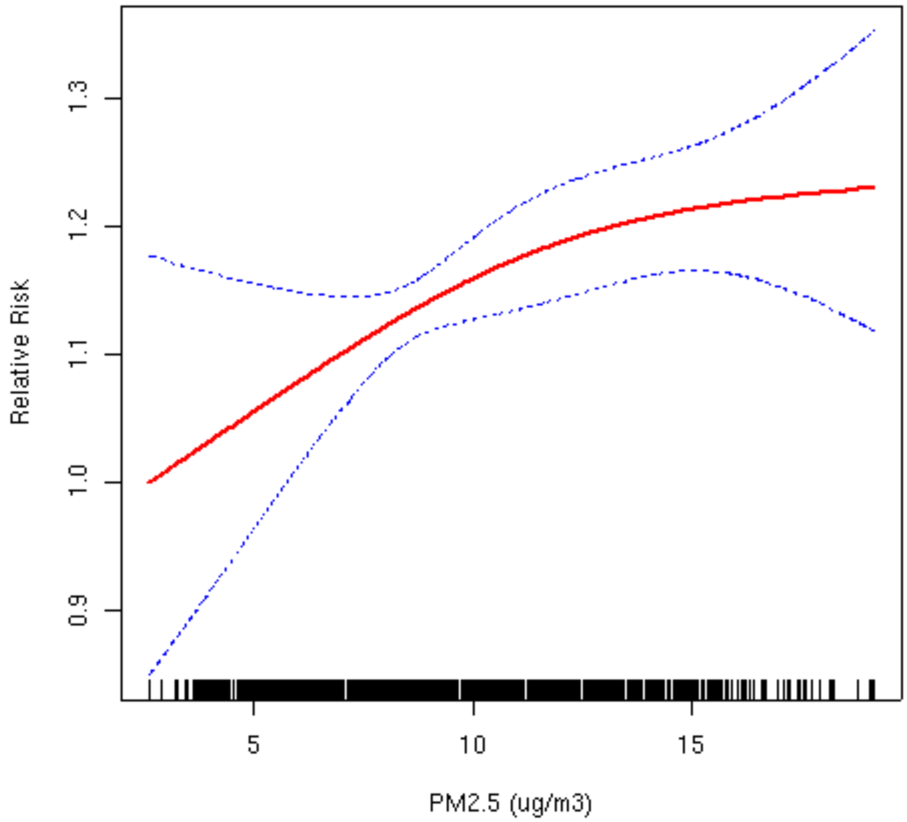

Supplemental Material, Figure S2. Concentration-response relationship between the concentration of $PM_{2.5}$ and incident diabetes among the cohort, depicted using a natural cubic spline function with 2 degrees of freedom. The hazard ratios were estimated by comparing to 2.6μg/m^3. The Cox model stratified by age, survey year and region, and adjusted for sex, marital status, education, household income, BMI, physical activity, smoking, alcohol consumption, diet, race, hypertension, urban residency, neighborhood-level unemployment rate, education, household income, and COPD, asthma, congestive heart failure, and acute myocardial infarction.

References

Austin PC, Daly PA, Tu JV. A multicenter study of the coding accuracy of hospital discharge administrative data for patients admitted to cardiac care units in Ontario. American Heart Journal. 2002;144:290-296.

Environment Canada. 2010. National Air Pollution Surveillance Network (NAPS). Available: http://www.ec.gc.ca/natchem/default.asp?lang=en&n=EE0E2169-1 [accessed 31 March 2011].

Gershon AS, Wang C, Guan J, Vasilevska-Ristovska J, Cicutto L, To T. Identifying individuals with physcian diagnosed COPD in health administrative databases. COPD. 2009;6:388-394.

Gershon AS, Wang C, Guan J, Vasilevska-Ristovska J, Cicutto L, To T. Identifying patients with physician-diagnosed asthma in health administrative databases. Can Respir J 2009;16:183-188.

Jerrett M, Burnett RT, Ma R, Pope CA, III, Krewski D, Newbold KB, et al. 2005. Spatial analysis of air pollution and mortality in Los Angeles. Epidemiology 16:727-736.

Lee DS, Austin PC, Rouleau JL, Liu PP, Naimark D, Tu JV. Predicting mortality among patients hospitalized for heart failure. JAMA. 2003;290:2581-2587.

Miller KA, Siscovick DS, Sheppard L, Shepherd K, Sullivan JH, Anderson GL, et al. 2007. Long-term exposure to air pollution and incidence of cardiovascular events in women. N Engl J Med 356:447-458.

Pope CA 3rd, Burnett RT, Thun MJ, Calle EE, Krewski D, Ito K, et al. 2002. Lung cancer, cardiopulmonary mortality, and long-term exposure to fine particulate air pollution. JAMA 287:1132-1141.

Tu JV, Naylor CD, Austin P. Temporal changes in the outcomes of acute myocardial infarction in Ontario, 1992-1996. CMAJ. 1999;161:1257-1261.

Tu JV, Austin PC, Walld R, Roos L, Agras J, McDonald KM. Development and validation of the Ontario acute myocardial infarction mortality prediction rules. J Am Coll Cardiol. 2001;37:992-997.

Tu K, Chen Z, Lipscombe LL. Prevalence and incidence of hypertension from 1995 to 2005: a population-based study. CMAJ. 2008;178:1429-1435.

Yeung DF, Boom NK, Guo H, Lee DS, Schultz SE, Tu JV. Trends in the incidence and outcomes of heart failure in Ontario, Canada: 1997 to 2007. CMAJ 2012;184:E765-E773
